# Challenges in acute management of cerebral sinovenous thrombosis among neonates with acute kidney injury: a retrospective cohort study

**DOI:** 10.3389/fstro.2025.1692460

**Published:** 2025-12-18

**Authors:** Manish Parakh, Marilyn Tan, Ankit Kumar Meena

**Affiliations:** 1 Dr S N Medical College, Jodhpur, India; 2 Dr Sampurnanand Medical College, Jodhpur, India; 3 University of the Philippines Manila, Manila, Philippines; 4 All India Institute of Medical Sciences Jodhpur, Jodhpur, India

**Keywords:** pediatric stroke, stroke management, systems of care, pediatric acute stroke protocols, CSVT

## Abstract

**Introduction:**

Cerebral sinovenous thrombosis (CSVT) in neonates with acute kidney injury (AKI) is a rare neurologic condition with potential serious consequences. Rapid diagnosis is key to good outcomes. This study aims to identify challenges in acute care and to evaluate outcomes of these patients in a resource-limited setting.

**Materials and methods:**

This retrospective cohort study included term neonates with AKI and CSVT admitted at a tertiary center in Western India (January 2021–January 2023). Clinical profile, timing of consult with healthcare providers, diagnosis, neuroimaging, management strategies, and outcomes at discharge and at age 2 years were analyzed.

**Results:**

A total of 31 neonates (19 male) with mean age 18.5 ± 6.6 days at diagnosis were included. Dehydration was the most common risk factor in 80.6%, while seizures were the most common clinical presentation (80.6% patients). Almost 84% of patients had thrombosis in multiple sinuses. Venous infarcts were identified in 20 (64.5%) patients, with concomitant hemorrhage in 13 (42%). Only 10 patients received anticoagulation therapy. Median time from symptom onset to consult in first healthcare facility was 48 h [interquartile range (IQR): 44–72 h]. Eighteen patients (58.06%) were subsequently referred to a second facility after a median stay of 48 h (IQR: 28–72 h). At the secondary or tertiary referral center, diagnostic neuroimaging was performed after a median of 48 h (IQR: 36–108 h). Anticoagulation was initiated within a median of 2 h (IQR: 2–2.75 h) following the diagnosis of CSVT. Although all patients survived, 32% had neurologic sequelae at discharge which persisted at the 2-year follow-up. Complete vessel recanalization on follow-up neuroimaging was achieved in all anticoagulated patients, compared with 66.7% of those who were not anticoagulated. However, statistical analysis showed no significant association between anticoagulation therapy and either clinical outcome or vessel recanalization.

**Conclusion:**

Neonatal CSVT associated with AKI can lead to persistent neurologic deficits at 2 years. Timely diagnosis and management remain a significant challenge in resource-limited settings due to delays both before and during hospitalization. Although anticoagulation treatment was not associated with outcomes in our cohort, further research is needed to develop acute care guidelines, applicable across diverse clinical settings, particularly in resource-limited situations.

## 1 Introduction

Cerebral sinovenous thrombosis (CSVT) is a rare neurologic condition in neonates, with an incidence of 1.4–12 per 100,000 term neonates per year. Risk factors are usually multifactorial and include dehydration, perinatal complications, congenital heart disease, sepsis, and prothrombotic states (Mandel-Shorer et al., 2022). In contrast, acute kidney injury (AKI) is relatively common among neonates in tropical countries, where hot and humid climates predispose individuals to dehydration. Neonates have a limited capacity to autoregulate renal blood flow, making them particularly vulnerable to glomerular and tubular injury (Cao et al., 2022). In a study by Gupta et al. (2024), 16% of neonates with AKI developed CSVT (Gupta et al., 2024). AKI reflects established renal injury which results in electrolyte/acid–base derangements, fluid imbalance, and altered drug clearances. These challenges are not uniformly present in uncomplicated dehydration. Additionally, uremia additionally causes neurologic symptoms and signs—making both diagnosis and anticoagulation decisions materially different from standard CSVT care pathways.

The clinical presentation of CSVT in neonates is often non-specific, and include lethargy, irritability, or seizures, which pose a challenge in early diagnosis and treatment. CSVT may result in death or long-term neurologic sequelae (Ferriero et al., 2019). In the study by Gupta et al. (2024), 12 neonates with CSVT and AKI were managed conservatively with correction of fluid, electrolyte and acid–base abnormalities, and anti-seizure medications (Gupta et al., 2024). None of them was anticoagulated, due to lack of guidelines and expertise in anticoagulation for CSVT with concomitant AKI. On discharge, abnormalities were noted in motor tone (25%), feeding (17%), and electroencephalography findings (33%) (Gupta et al., 2024). Published pediatric stroke guidelines state that anticoagulation may be considered in neonates with CSVT, particularly in the presence of clinical deterioration or thrombus propagation on serial imaging (Ferriero et al., 2019). The lack of specialists experienced in anticoagulating neonates having associated comorbidities such as AKI, along with the limited availability and high cost of anticoagulants, remain major challenges in management, especially in resource-limited settings.

Several studies have examined diagnostic delays and prehospital care in childhood arterial ischemic stroke (AIS) (Rafay et al., 2009; Srinivasan et al., 2009; Mallick et al., 2015; Stojanovski et al., 2017; Pai et al., 2024), but none have addressed this issue in neonatal CSVT. This study was therefore undertaken to identify the challenges in diagnosing and acutely managing CSVT among neonates with AKI in a regional tertiary care center caring for both inborn and outborn neonates with level III neonatal intensive care units. In addition, it aims to identify predictors of anticoagulant use and to determine the association between anticoagulant use and outcomes.

## 2 Materials and methods

This retrospective cohort study included term neonates (37–41 weeks) appropriate for gestational age (0–28 days old) with both AKI and CSVT who were admitted to a tertiary care center in Western India between January 2021 and January 2023. AKI was labeled as per Kidney Disease: Improving Global Outcome (KDIGO) criteria. Neonates with congenital malformations, perinatal asphyxia, hypoxic–ischemic injury from other causes, or central nervous system (CNS) infection were excluded, as were those born to mothers with medical conditions such as pregnancy-induced hypertension, eclampsia, antepartum hemorrhage, peripartum infections, gestational diabetes mellitus, hyperemesis gravidarum, and also those with abnormal fetal Doppler findings. Although this reduced the sample size, it allowed for a clearer assessment of the clinical features, management, and outcomes specific to AKI-associated CSVT. This focused approach minimized bias that could arise from conditions that independently affect AKI or its presentation.

Patient medical records were reviewed after obtaining approval from the institutional ethics board. The demographic characteristics of each patient, including gestational age, sex, birth weight, and admission weight, were documented. Clinical features such as age at symptom onset and at CSVT diagnosis, presenting symptoms, CSVT risk factors, cranial MRI/MRV findings, and treatment received were also recorded. Additional data collected included the time interval from symptom onset to consultation at the first health care facility, the interval from the first facility to arrival at the referral (secondary/tertiary) facility, and the interval from arrival to diagnostic neuroimaging, along with causes of delay. Symptom onset was considered as the first caregiver-reported occurrence of any new symptom(s) that continued as part of the same clinical syndrome until CSVT was diagnosed. The interval from symptom onset to first healthcare consultation was calculated using this operational definition.

Challenges in acute management were also evaluated, including the time interval from hospital admission to treatment initiation and factors influencing management such as availability, cost, monitoring, and expertise with anticoagulation. Short-term outcomes at discharge and long-term neurodevelopmental outcomes at 2 years of age were assessed and compared between neonates who received anticoagulation and those who did not.

Descriptive statistics summarized clinical and time interval data. Continuous variables were expressed as median with interquartile range (IQR). Group comparisons were performed using Student's *t*-test, Welch's *t*-test, Fisher's exact test (*n* ≤ 5), chi-square test, or Mann–Whitney test, as appropriate. Statistical significance was set at *p* < 0.05. Logistic regression was used to identify independent predictors of anticoagulant use and its association with outcomes.

## 3 Results

### 3.1 Demographic and clinical profile of neonates with CSVT and AKI

A total of 31 term neonates (19 male, 12 female) were included in the study. The mean gestational age at birth was 38.22 ± 1.06 weeks, and the mean birth weight was 2,653.22 ± 280.49 g. The mean age at symptom onset was 12.67 ± 6.67 days, and the mean age at CSVT diagnosis was 18.51 ± 6.63 days. In the overall cohort, 24/31 (77.4%) were from rural areas and 7/31 (22.6%) from urban areas; among anticoagulated neonates, 80.0% were rural and 20.0% urban, while among those not anticoagulated, 76.2% were rural and 23.8% urban. Dehydration was the most common risk factor, present in 80.6% of cases, followed by infection (sepsis or CNS infection) in 38.7% of cases. Neonates with confirmed sepsis or CNS infection at enrollment were excluded; however, during hospitalization, some infants developed clinical features concerning for intercurrent sepsis/CNS infection and were treated empirically with antibiotics despite normal cerebrospinal fluid (CSF) studies and negative microbial cultures, and these episodes were recorded as “suspected sepsis during hospitalization” (Table 1).

**Table 1:** Clinical profile of neonates with cerebral sinovenous thrombosis and acute kidney injury.

**Clinical, radiologic and treatment characteristics**	**All patients *n* = 31 (%)**	**ACT *n* = 10 (%)**	**No ACT *n* = 21 (%)**	***p*-Value**
**Patient characteristics**				
Male	19	8	11	
Mean gestational age at birth (weeks)	38.22 ± 1.05	38.0 ± 0.82	38.19 ± 1.16	0.600
Mean birth weight (grams)	2,653.22 ± 280.49	2,875 ± 235.05	2,547.61 ± 235.05	0.001
Mean weight at CSVT diagnosis (grams)	2,316.45 ± 446.11	2,695.0 ± 417.51	2,136.19 ± 338.10	0.003
Mean age at symptom onset (days)	12.67 ± 6.67	17.30 ± 8.05	10.47 ± 4.66	0.028
Mean age at CSVT diagnosis (days)	18.51 ± 6.63	23.90 ± 6.69	15.95 ± 4.95	0.001
**Risk factors**				
Suspected sepsis	12 (38.7%)	0 (0%)	12 (57.1%)	0.003
Dehydration	25 (80.6%)	8 (80%)	17 (81%)	1.000
Anemia	6 (19.4%)	0 (0%)	6 (28.6%)	1.000
Thrombocytopenia	11 (35.5%)	2 (20%)	9 (42.8%)	0.270
**Acute clinical presentation**				
Seizures	25 (80.6%)	9 (90%)	16 (76.2%)	0.630
Decreased sensorium	21 (67.7%)	6 (60%)	15 (71.4%)	0.710
**Sinus involvement**				
>1 sinus	26 (83.9%)	6 (60%)	20 (95.2%)	0.028
Superficial	7 (22.6%)	3 (30%)	4 (19.0%)	0.640
Deep	17 (54.8%)	7 (70%)	10 (47.6%)	0.290
Combined	7 (22.6%)	0 (0%)	7 (33.3%)	0.061
**Cranial MRI findings**				
Venous infarcts	20 (64.5%)	7 (70%)	13 (61.9%)	0.710
Hemorrhage	13 (41.9%)	1 (10%)	12 (57.1%)	0.020
**Treatment**				
Anticoagulant (heparin)	10 (32.2%)	10 (100%)	0 (0.00%)	0.001
Inotropes	8 (25.8%)	0 (0%)	8 (38.1%)	0.026
Antibiotics	13 (41.9%)	0 (0%)	13 (61.9%)	0.001
Anti-seizure medications	20 (64.5%)	3 (30%)	17 (81%)	0.008

ACT, anticoagulant treatment.

Anticoagulation was administered to 10 of 31 neonates (32.3%), of whom eight were male. Compared with those who did not receive anticoagulation, these neonates had significantly higher birth weights and admission weights, and they were significantly older at both symptom onset and the time of CSVT diagnosis (Table 1).

Seizures were the most common clinical presentation, occurring in 80.6% of patients, followed by decreased sensorium in 67.7%. Multiple sinus involvement was observed in 83.9% (*n* = 26), with the majority (*n* = 20) not receiving anticoagulation. Cranial MRI revealed venous infarcts in 64.5% (*n* = 20) of cases, of which 13 had concomitant hemorrhage. Notably, 92% of neonates with hemorrhage were not anticoagulated. In addition, none of the patients receiving inotropes (*n* = 8) or antibiotics (*n* = 13) were anticoagulated, and only three of 20 patients on anti-seizure medications received anticoagulant therapy (Table 1).

### 3.2 Challenges in diagnosis and acute management of CSVT in neonates with AKI

#### 3.2.1 Time from symptom onset to consult in first healthcare facility

The median interval from symptom onset to consultation at the first healthcare facility for all patients was 48 h (IQR: 44–72 h), with no significant difference between anticoagulated and non-anticoagulated patients. Twenty patients (64.5%) first sought consult in tertiary referral centers where pediatricians, neonatologists, or pediatric neurologists were available at any point in time. Nine of these 20 patients received anticoagulation. Four patients (12.9%) first sought care at secondary-level community health centers, with only one receiving anticoagulation. Three patients (9.7%) first presented to primary healthcare centers manned by primary care physicians or MBBS internists, and four (12.9%) to sub-centers managed by nursing personnel or midwives. None of the patients in these latter two groups received anticoagulation therapy (Table 2).

**Table 2:** Time intervals from symptom onset to diagnosis and acute management.

**Median time**	**All patients *n* = 31 median (IQR)**	**ACT *n* = 10 median (IQR)**	**No ACT *n* = 21 median (IQR)**
Time from symptom onset to consult in first healthcare facility (hours)	48 (44–72)	72 (34.5–100.5)	48 (44–60)
Duration of stay in the first healthcare facility if referred to another facility for further management (hours)	48 (28–72) (*n* = 18)	22 (*n* = 1)	48 (28–72) (*n* = 17)
Time before availability of transportation to tertiary referral center (hours)	12 (8–26)	3 (3–3)	18 (12–27)
Time from request by specialist to performance of diagnostic neuroimaging after arrival in secondary/tertiary referral center (hours)	48 (36–108)	38 (24.75–48)	96 (48–120)
Time from request by specialist to performance of neuroimaging (hours)	24 (23–72)	24 (7–24)	48 (24–90)
Time to initiation of acute supportive management (minutes)	10 (10–25)	18 (10–25)	10 (10–15)
Time to initiation of anticoagulation treatment (hours)	—	2 (2–2.75)	—
Duration of hospital stay (days)	8 (8–10.5)	8 (7–9.5)	9 (8–14)
Time to repeat neuroimaging after discharge (weeks)	7 (6–8)	7 (6.25–10)	8 (6–8)

ACT, anticoagulant treatment.

The first healthcare provider encountered by 21 patients (67.7%) was a pediatric neurologist or pediatrician. All children (*n* =10) who received anticoagulation were reviewed and managed at a secondary or tertiary center with the expertise of a pediatric neurologist/pediatrician. Six patients (19.4%) initially consulted a primary care physician or MBBS internist, while four (12.9%) were first seen by a nurse or auxiliary nurse midwife; none of the patients in these latter groups received anticoagulation.

#### 3.2.2 Time from consult in first healthcare facility to arrival in second healthcare facility

Eighteen of 31 patients (58.1%) were referred to a second healthcare facility after a median stay of 48 h (IQR: 28–72 h) at the initial center. Only one patient received anticoagulation following referral, having been transferred after 22 h. The remaining 17 patients, who were referred after a longer median stay of 48 h (IQR: 28–72 h), did not receive anticoagulation.

The main reasons for referral included deteriorating clinical condition and lack of neuroimaging facilities (*n* = 15), inadequate infrastructure or equipment for managing critically ill neonates (*n* = 11), and unavailability of trained pediatric specialists (*n* = 6). Once referral was initiated, the median time to transfer was 12 h (IQR: 8–26 h) (Table 2).

#### 3.2.3 Time to diagnostic neuroimaging

Upon arrival at a secondary or tertiary referral center, the median time to cranial MRI/MRV was 48 h (IQR: 36–108 h). This occurred after a median of 24 h (IQR: 23–72 h) from the time the study was requested by a specialist for suspected CSVT. Reported reasons for delays in neuroimaging included unstable clinical condition making transport difficult (*n* = 30), absence of standardized neuroimaging protocols for neonates with AKI (*n* = 29), unavailability of anesthesiologists or specialists for sedation (*n* = 19), and a low index of suspicion for CSVT (*n* = 14). Both the time from arrival to neuroimaging and the time from requisition to study completion were significantly shorter in patients who received anticoagulation (*p* < 0.001 and *p* = 0.002, respectively). Notably, only 13 patients were diagnosed with CSVT at the first healthcare facility, all of whom had presented directly to our hospital (Table 2).

#### 3.2.4 Factors associated with time to radiologic confirmation of CSVT

In univariate analysis, factors associated with time to CSVT diagnosis included encephalopathy at presentation, duration of symptoms before first consultation, type of healthcare provider first encountered, and referral to a second facility. Seizure was not significant in univariate analysis but was included in the multivariate model given its clinical relevance, as this symptom often prompts parents to seek medical attention. In multivariate analysis, seizure at presentation emerged as the only significant factor associated with time to CSVT diagnosis (*p* < 0.001) (Table 2).

#### 3.2.5 Time from hospital admission to treatment initiation

Acute management, including fluids, inotropes, antibiotics, and anti-seizure medications as indicated, was initiated promptly after hospital admission, with a median time of 10 min (IQR: 10–25 min). This did not differ significantly between patients who received anticoagulation and those who did not. Among the 10 patients who received anticoagulants, treatment was started within a median of 2 h (IQR: 2–2.75 h) from the time of CSVT diagnosis, and no post-treatment hemorrhage was detected on follow-up neuroimaging. The most frequently cited reasons for withholding anticoagulation in the remaining 21 patients were unavailability of laboratory facilities and clinical expertise (*n* = 20), absence of standardized guidelines for CSVT with associated AKI (*n* = 21), and concomitant CNS infection (*n* = 18) (Table 2).

#### 3.2.6 Predictors of anticoagulant use

In univariate analysis, predictors of antithrombotic use included age at symptom onset, presence of hemorrhage, referral to another facility, and time to diagnostic neuroimaging. Age and hemorrhage were no longer significant in multivariate analysis, and multivariate modeling could not be performed for referral and neuroimaging delay. Although duration of hospital stay was not significant in univariate analysis, it was included in the multivariate model given its clinical relevance as a marker of illness complexity or severity. In this analysis, longer hospital stay was significantly associated with reduced antithrombotic use, with odds of anticoagulation decreased by 60% (OR 0.4, 95% CI: 0.18–0.97, *p* = 0.04) (Table 2).

### 3.3 Outcomes of neonates with CSVT and AKI

#### 3.3.1 Clinical outcome

All 31 patients survived and were discharged home. At discharge, 21 patients (67.7%) were asymptomatic with normal neurological examination, while 10 patients (32.3%) had persistent symptoms such as excessive crying, seizures, or feeding difficulties, along with abnormal neurological findings including altered sensorium, tone abnormalities, and hyperreflexia. Out of these 10 patients, 3 (30%) had received anticoagulation and 7 (70%) had not. Multiple sinus thrombosis was present in eight of these 10 infants (80%), indicating that persistent symptoms may be more common with greater radiologic burden of venous involvement. At 2-year follow-up, abnormal neurodevelopment was documented in 10 children; out of these, 3 (30%) had been anticoagulated and 7 (70%) had not, and multiple sinus thrombosis was identified in 8 of 10 (80%), indicating that multi-sinus involvement may have adverse clinical and developmental outcomes over time. The median duration of hospital stay was 8 days (IQR: 8–10.5 days), with no significant difference between those who received anticoagulation and those who did not.

At 2-year follow-up, 21 patients (67.7%) demonstrated normal neurodevelopment and neurological examination, whereas 10 patients (32.3%) showed abnormal neurodevelopment. The Pediatric Stroke Outcome Measure revealed deficits including cerebral palsy with global developmental delay in five patients and language delay with hyperactivity in five patients. Clinical outcomes, both at discharge and at 2-year follow-up, did not differ significantly between patients who received anticoagulation and those who did not (Table 3).

**Table 3:** Outcomes of neonates with cerebral sinovenous thrombosis and acute kidney injury.

**Outcome**	**All patients *n* = 31(%)**	**ACT *n* = 10 (%)**	**No ACT *n* = 21 (%)**	***p*-Value**
Discharged alive	31 (100%)	10 (100%)	21 (100%)	
**Status at discharge**				
Asymptomatic with normal neurologic examination	21 (67.7%)	7 (70%)	14 (66.7%)	1.000
Symptomatic with or without abnormal neurologic examination	10 (32.2%)	3 (30%)	7 (33.3%)	1.000
**Outcome at 2 years**				
Normal neurodevelopment with normal neurologic examination	21 (67.7%)	7 (70%)	14 (66.7%)	1.000
Abnormal neurodevelopment with or without abnormal neurologic examination	10 (32.2%)	3 (30%)	7 (33.3%)	1.000
**Radiologic outcome**				
Complete vessel recanalization on repeat neuroimaging after discharge	24 (77.4%)	10 (100%)	14 (66.7%)	0.041

ACT, anticoagulant treatment.

#### 3.3.2 Radiologic outcome

Repeat neuroimaging was performed in all patients at a median of 7 weeks after discharge (IQR: 6–8 weeks). However, cranial MRI data were available for review in only 23 patients. Of the 20 patients with venous infarcts on initial imaging, seven had received anticoagulation while 13 had not. Among the seven anticoagulated patients, repeat MRI data were available for only three, all of whom showed persistence of parenchymal abnormalities. Of the 13 patients who did not receive anticoagulation, six showed resolution of parenchymal abnormalities, six had persistent lesions, and data were unavailable for one patient. The limited availability of follow-up MRI in nearly half of the anticoagulated patients makes direct comparison of parenchymal resolution between groups difficult.

In contrast, follow-up MRV demonstrated complete recanalization in 24 of 31 patients (77.4%). All 10 patients who received anticoagulation achieved complete recanalization, compared with 66.7% of those who were not anticoagulated (Table 3).

#### 3.3.3 Association of anticoagulant use with outcome

Although the proportion of patients with complete recanalization was higher among those who received anticoagulation, regression analysis showed that anticoagulant use was not a significant predictor of vessel recanalization. Similarly, anticoagulant use was not associated with clinical outcomes.

## 4 Discussion

### 4.1 Clinical profile of neonates with CSVT and AKI

Similar to the International Pediatric Stroke Study (IPSS) cohort (Jordan et al., 2010), our study also reported seizures and altered sensorium as the two most common clinical presentations of CSVT, regardless of ethnicity. In contrast, focal motor deficits were not observed in our cohort but were reported in 6% of patients in the IPSS cohort. Most patients in the IPSS cohort presented during the first postnatal week, whereas symptom onset in our cohort typically occurred during the second to third week of life. This difference may be explained by the higher frequency of peripartum risk factors and the inclusion of mothers with comorbidities in the IPSS group, while our cohort was restricted to neonates with AKI and excluded those with maternal comorbidities. Consequently, dehydration was a very common risk factor in our cohort (80.7%) compared with only 13% in the IPSS cohort. Venous infarcts and hemorrhage were common MRI findings in both groups. The use of heparin was higher in the IPSS cohort (59.2%) compared with our cohort (32.2%), likely reflecting greater availability of stroke specialists and expertise in anticoagulation at participating IPSS centers in 2010.

### 4.2 Challenges in diagnosis and acute management of CSVT in neonates with AKI

#### 4.2.1 Time from symptom onset to consult in first health care facility

The current study observed that patients sought consultation at a healthcare facility only after a median of 48 h from symptom onset, which is considerably longer than the median of 1 h reported in children with AIS (Rafay et al., 2009). The abrupt onset of symptoms in AIS likely prompts more urgent consultation, in contrast to the more insidious onset of CSVT. However, the median time to AIS diagnosis in neonates is significantly longer than in older children due to the absence of localizing signs (Srinivasan et al., 2009). Because neonatal CSVT symptoms are frequently non-specific and evolve insidiously, caregiver recall of exact onset may be imprecise. Our operational definition of “symptom onset”—first persistent new symptom(s) reported by caregivers—may introduce recall bias and misclassification; nonetheless, it allowed standardized time interval estimates aligned with the study's focus on diagnostic and therapeutic delays in CSVT with AKI. Thus, the non-specific nature of symptoms and the lack of localizing signs in neonates as a group are the most likely reasons for delayed healthcare consultation and delayed stroke diagnosis, both in CSVT and AIS.

Although 64.5% of our patients were first brought to tertiary referral centers, three patients (9.7%) were initially taken to primary healthcare centers and four patients (12.9%) to sub-centers. In these settings, CSVT diagnosis was not established, and anticoagulation therapy could not be initiated. The proximity of these healthcare centers, and sub-centers made it more accessible to patients but resulted in longer time to diagnosis and treatment initiation.

#### 4.2.2 Time from consult in first health care facility to arrival in second health care facility

Fifty-eight percent of patients were referred to a second health care facility after a median stay of 48 h (IQR: 28–72 h) at the initial health care facility with only one patient subsequently receiving anticoagulation, having been referred to the second facility after 22 h. The remaining patients stayed longer in the first health care facility with median stay of 48 h (IQR: 28–72 h) before transfer and did not receive anticoagulation. Once referral was initiated, transportation to the second facility became available after a median of 12 h (IQR: 8–26 h).

#### 4.2.3 Time to diagnostic neuroimaging

Another challenge in establishing the diagnosis was the delay in performing diagnostic neuroimaging. Cranial MRI/MRV was available only in secondary and tertiary healthcare facilities, whereas cranial ultrasound was the sole imaging modality in smaller centers. In referral centers, cranial MRI/MRV was performed after a median of 48 h (IQR: 36–108 h) from arrival, and after a median of 24 h (IQR: 23–72 h) from the time it was requested by a specialist for suspected CSVT.

#### 4.2.4 Factors associated with time to radiologic confirmation of CSVT

Although the type or expertise of the first healthcare provider encountered and referral to a second facility were associated with time to neuroimaging diagnosis of CSVT in univariate analysis, seizure at presentation was the only variable that remained significant in multivariate analysis (*p* < 0.001). The abrupt onset of seizures likely prompted parents to seek earlier consultation and facilitated more timely assessment by physicians.

#### 4.2.5 Time from hospital admission to treatment initiation

After diagnosis of CSVT has been made, the next challenge lies in management. Although acute supportive management which included fluids, inotropes, antibiotics, or anti-seizure medication as necessary was initiated promptly after hospital admission at a median of 10 min (IQR: 10–25 min), only 10 patients (32.2%) were given anticoagulants. Nine of these patients were treated in tertiary health care facilities and one in a secondary health care facility. Reasons cited for not giving anticoagulants in the 21 remaining patients were unavailability of lab facilities and clinical expertise, lack of guidelines for concomitant AKI, and presence of CNS infection.

In a landmark prospective study, Moharir et al. (2010) demonstrated that anticoagulant therapy (ACT) in pediatric CSVT appears safe, with thrombus propagation occurring significantly more frequently in untreated patients (28% of neonates and 37% of children) than in those receiving anticoagulation (4 and 7%, respectively). Propagation was associated with new venous infarcts and adverse clinical outcomes, particularly in children, suggesting that while ACT carries a small hemorrhage risk, the benefit of thrombus arrest and prevention of secondary infarction may outweigh this risk in carefully selected cases. However, Moharir's cohort (1992–2009) predominantly comprised term infants with mixed-etiology CSVT managed in a quaternary-care setting, and did not separately analyze the subgroup of neonates with concurrent AKI—a population in which both hemorrhage risk and anticoagulation drug clearance may be substantially altered. Our study addresses this evidence gap by examining neonatal CSVT specifically in the context of AKI, where the absence of established protocols and robust data compounds the clinical dilemma regarding anticoagulation decisions. The high prevalence of multiple sinus thrombosis among our infants with persistent or abnormal neurodevelopmental outcomes underscores the potential gravity of more extensive cerebral thrombotic disease in this population; nonetheless, the optimal strategy for patient selection, anticoagulation dosing, and hemorrhage surveillance in neonates with CSVT and concurrent AKI remains unresolved and observations from the current study will help formulate management guidelines in these complex clinical situations.

#### 4.2.6 Predictors of anticoagulant use

In the current study, clinical notes from bed head tickets did not indicate any formal weight-based criteria for anticoagulation, yet anticoagulated infants tended to be larger or older, suggesting that physicians may be hesitant to anticoagulate younger neonates with lower weight, given perceived bleeding risks and narrower therapeutic windows in neonates with CSVT and concomitant AKI. These interpretations are inferential and warrant confirmation through larger, prospective studies that capture clinician decision rationales directly.” Intracranial hemorrhage was not a predictor of anticoagulant treatment or non-treatment in the current cohort, which was similar to the observations made in the IPSS cohort (Jordan et al., 2010). Instead, duration of hospital stay indicative of illness complexity or severity, was a significant predictor of antithrombotic use with odds of anticoagulant use (OR 0.4, 95% CI: 0.18–0.97, *p* = 0.04) reduced by 60% in patients with longer hospital stays. During hospitalization, 12/31 (38.7%) neonates were suspected to have sepsis on the basis of clinical picture. However, as stated above, they had normal CSF studies and negative microbial culture and none received anticoagulation. It is likely that clinicians may have avoided anticoagulation in these neonates because of suspected infection with AKI, posing a potential challenge in managing and monitoring anticoagulation.

While the median interval from symptom onset to initial consultation and the duration of stay at the first healthcare facility did not differ significantly between anticoagulated and non-anticoagulated patients, the median time from arrival at a secondary or tertiary center to performance of neuroimaging, as well as the interval from requisition to completion of neuroimaging, were both significantly shorter in patients who received anticoagulation. This suggests that, although prehospital delays were present, the more critical delay in confirming CSVT diagnosis and initiating treatment was attributable to in-hospital processes and delays.

In the current study, almost two-thirds of neonates did not receive anticoagulation, mostly attributed to lack of expertise and guidelines to deal with specific complex situations, for example, CSVT with AKI. Moreover, in our setting, diagnostic delays mostly reflect interacting socioeconomic and health-system determinants, including access to the healthcare system, distance from tertiary centers, referral pathways through multiple facilities before neuroimaging, and limited availability of advanced MRI/MRV and trained specialists in peripheral hospitals, all of which can defer definitive evaluation for suspected neonatal CSVT especially when complicated with AKI. All of these factors need further studies to determine regional resource and access constraints surrounding neonatal CSVT recognition and work-up, particularly where symptoms are non-specific and pre-transport stabilization, transport logistics, and timely imaging access can each add substantial delays in diagnosis and treatment.

Anticoagulation guidelines are not well-established for complex situations, and further studies are needed to develop guidelines for these specific situations, especially in a neonate or young infant. This study was conducted in public hospitals in Western India where anticoagulants are provided without charge. Socioeconomic factors are important in health care access and utilization throughout the world and may influence optimal management in all settings. In the current study, treatment decisions for CSVT with concomitant AKI were influenced by protocol considerations rather than direct patient costs. Although socioeconomic status was not collected here, residence data show a predominantly rural cohort. Future studies are required to evaluate socioeconomic factors and health-system access indicators to better delineate equity impacts on neonatal CSVT care and outcomes.

### 4.3 Outcomes of neonates with CSVT and AKI

Although all patients survived and more than half achieved good short- and long-term outcomes, 32% developed neurological sequelae both at discharge and at 2-year follow-up. Clinical outcomes at both time points did not differ significantly between patients who received anticoagulation and those who did not. In contrast, complete recanalization on repeat cranial MRV was observed in 24 of 31 patients (77.4%), with 100% recanalization in all 10 anticoagulated patients compared to 66.7% in the non-anticoagulated group.

By comparison, the Canadian single-center cohort reported a markedly less favorable clinical profile, with 59% of neonates experiencing unfavorable outcomes and 85% achieving recanalization at 3 months. In their analysis, lack of anticoagulation was predictive of unfavorable outcome in univariate analysis, but anticoagulation was not associated with recanalization. On multivariate analysis, lack of anticoagulation was no longer a significant predictor of outcome (Moharir et al., 2010). Regression analysis in our study yielded similar findings.

A recent meta-analysis, however, demonstrated that anticoagulation therapy in neonatal CSVT was associated with a reduced risk of thrombus propagation (risk ratio 0.14, 95% CI: 0.03–0.72), without evidence of increased morbidity or mortality. These findings highlight a position of equipoise and underscore the need for well-designed randomized controlled trials to clarify the role of anticoagulation in neonatal CSVT (Rossor et al., 2018).

## 5 Conclusion

Our study showed that neonatal CSVT can result in significant neurologic deficits that persist at 2 years, but timely diagnosis and management of CSVT remain a significant challenge in resource-limited settings. Although prehospital delays due to health-seeking behavior, lack of trained specialists, and limited access to advanced neuroimaging in peripheral centers with limited transportation to tertiary care facilities exist, the current study showed that there was a statistically significant association between in-hospital delays and time to CSVT diagnosis and anticoagulation treatment. The main reason for delays in neuroimaging in the current study was the fact that many neonates were too critically ill to be transported to the MRI suite. It is therefore recommended to enhance the skills of healthcare providers in performing bedside cranial ultrasound for the detection of CSVT, and to advocate its use as an alternative to MRI/MRV in unstable patients, especially in resource-limited settings. These potentially modifiable causes of delay underscore the need to increase disease awareness among members of the community and healthcare providers, and the need for system-wide interventions to improve the triage system, access to neuroimaging, and capacity for anticoagulation treatment in peripheral centers. In our public hospital setting, treatment decisions for CSVT with concomitant AKI were driven by clinical decisions rather than patient costs, despite the predominantly rural background of the cohort. Although socioeconomic status related to anticoagulation and imaging were not directly measured, these factors may still influence access and outcomes. Future studies should evaluate socioeconomic and health-system determinants to clarify equity impacts on neonatal CSVT care in complex situations. Although anticoagulation treatment was not associated with outcome in our study, probably due to the small sample size, we support the recommendation for well-designed randomized controlled trials in neonatal CSVT.

## 6 Study limitations and future directions

Our study is limited by its relatively small cohort from a single center and the inherent bias in treatment allocation due to resource constraints. The lack of standardized treatment protocols and variable follow-up durations may also influence the interpretation of long-term outcomes. The main limitation of this study is counterbalanced by the unique contribution it makes, as this is the first to explore challenges in acute care and outcomes of neonatal CSVT with AKI. To our knowledge, while diagnostic delays have been examined in AIS, no prior studies have specifically addressed such delays in neonatal CSVT. In addition, these findings highlight the urgent need for multicenter studies and the development of pragmatic guidelines for the management of neonatal CSVT in AKI applicable in various resource-limited settings including low- and middle-income countries.

## Data Availability

The original contributions presented in the study are included in the article/supplementary material, further inquiries can be directed to the corresponding author.
